# Population Bottlenecks Promote Cooperation in Bacterial Biofilms

**DOI:** 10.1371/journal.pone.0000634

**Published:** 2007-07-25

**Authors:** Michael A. Brockhurst

**Affiliations:** School of Biological Sciences, University of Liverpool, Liverpool, United Kingdom; Lund University, Sweden

## Abstract

Population bottlenecks are assumed to play a key role in the maintenance of social traits in microbes. Ecological parameters such as colonisation or disturbances can favour cooperation through causing population bottlenecks that enhance genetic structuring (relatedness). However, the size of the population bottleneck is likely to play a crucial role in determining the success of cooperation. Relatedness is likely to increase with decreasing bottleneck size thus favouring the evolution of cooperation. I used an experimental evolution approach to test this prediction with biofilm formation by the bacterium *Pseudomonas fluorescens* as the cooperative trait. Replicate populations were exposed to disturbance events every four days under one of six population bottleneck treatments (from 10^3^ to 10^8^ bacterial cells). In line with predictions, the frequency of evolved cheats within the populations increased with increasing bottleneck size. This result highlights the importance of ecologically mediated population bottlenecks in the maintenance of social traits in microbes.

## Introduction

Explaining the evolution of cooperation in microbes represents a major challenge [1], [2], [3], [4]. The central problem is that cooperation is often metabolically costly to individuals thus, all else being equal, cheats that reap the rewards of cooperation without making any investment should be able to invade populations of cooperators [5]. A classic solution to this problem is kin-selection [6], [7], [8]; here cooperation can be favoured if the benefits of cooperative acts are directed towards kin with whom the cooperator shares genes. Thus mechanisms that lead to cooperators interacting more with each other than with cheats (high relatedness) can favour the evolution of cooperation. Among metazoans, behavioural mechanisms such as kin-recognition may play a key role [9], [10], but kin recognition in microbes is far more contentious. As such, ecological conditions that increase spatial proximity of kin are likely to influence the evolution of microbial social traits.

Which conditions will result in increased spatial proximity of kin? First, if dispersal is limited, aggregations of close kin are likely to develop [11], [12], [13]. However, limited dispersal can also result in close relatives competing more with each other, which can result in selection against cooperation [14]. Second, ecological parameters such as metapopulation dynamics [1] (i.e., colonisation and extinction) and disturbances [15] can lead to increased genetic structuring (relatedness) if clonal or relatively few individuals found new populations; this causes population bottlenecks and potentially leads to the purging of cheats at regular intervals. A previous study has shown that very extreme population bottlenecks can have this effect [16]: In metapopulations seeded with distinct cheat and cooperator genotypes, either 1 or 2 *Pseudomonas aeruginosa* clones were used to found new patches. Higher frequencies of cooperation were observed with the smaller bottleneck, or in other words, under higher relatedness. However, the role of a broad range of population bottlenecks has yet to be considered; it is likely that the frequency of cooperation will increase with decreasing population bottleneck size. Moreover it is valuable to test the evolutionary effects of population bottlenecks in experimental systems where social traits evolve *de novo* [15], [17], [18], [19], [20], rather than more simple ecological competition between cooperator and cheat genotypes [16], [21].

I investigated the impact of population bottleneck size on the evolution of cheats experimentally using the cooperative trait of biofilm formation in *Pseudomonas fluorescens* [20], [22]. When propagated in spatially heterogeneous environments (a static glass microcosm containing nutrient-rich medium [22]), populations of the ancestral smooth (SM) *P. fluorescens* genotype rapidly diversify, generating a range of niche specialist genotypes by mutation that are maintained by negative frequency dependent selection [22]. The wrinkly-spreader (WS) morph is ecologically dominant [23], [24], forming a biofilm at the air-broth interface through constitutive overproduction of cellulosic polymer [25]. While over-expression of cellulosic polymer is individually costly (as demonstrated by the reduced exponential growth rate of WS relative to SM [20], [26]), its production provides a group benefit to WS because colonisation of the air-broth interface niche allows improved access to oxygen, a limiting resource [20]. Clonal WS biofilms have been found to be susceptible to invasion by SM genotypes that arise by mutation from WS [15], [18], [20]. In this context SM are cheats, gaining the benefit of inhabiting the air-broth interface while making no contribution to the integrity of the biofilm, which is significantly weaker in the presence of cheating SM genotypes [20].

Four independent WS genotypes were isolated from separate adaptive radiations. Initially isogenic populations of each were then propagated under a disturbance frequency that has previously been shown to favour cooperation (once every four days [15]). Disturbances were non-specific mass mortality events that varied in size between treatments: after thorough homogenisation, either 0.00001%, 0.0001%, 0.001%, 0.01%, 0.1%, 1%, or 10% of each population was transferred to a fresh microcosm. Experiments were performed in static microcosms over a 16-day period after-which-time populations were homogenised, plated onto agar and the frequency of SM and WS colonies determined. It was hypothesised that the frequency of evolved SM cheats would increase with increasing bottleneck size.

## Methods

### a. Isolating WS genotypes

Four replicate microcosms (30mL glass universal containing 6mL of King's B nutrient media) were inoculated with *Pseudomonas fluorescens* SBW25 to a total of approximately 10^7^ cells. These were statically incubated for 6 days at 28°C, after which time all populations were vortexed and an aliquot diluted and plated onto KB agar. A single wrinkly-spreader colony was then isolated from each population for further study and stored at −80°C in 20% glycerol.

### b. Population bottleneck selection experiment

Populations were initiated with 10^7^ cells of one of the isolated WS genotypes grown for 18h under shaken conditions. These were then propagated under one of the following population bottleneck regimes regimes: 0.006, 0.06, 0.6, 6, 60 or 600 µL of culture was transferred to a fresh microcosm every 4 days over a 16-day period. After 16 days the populations were homogenised and sampled. Samples were then plated onto agar and the frequencies of WS and SM colonies counted. Data of the proportion of WS cooperators was arcsin square-root transformed to stabilize variance and analysed in a General Linear Model fitting log_10_ bottleneck size as a covariate and founding population as a random factor. Log_10_ transformed total population density data was analysed in the same way. Both analyses conformed to GLM assumptions of normality of residuals and homogeneity of variance.

## Results and Discussion

**Figure 1:**
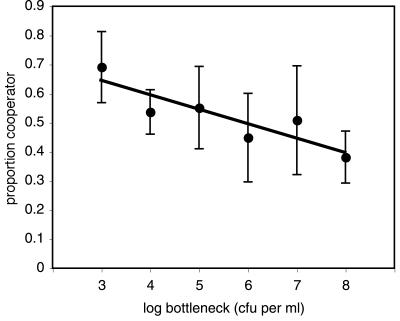
The effect of population bottleneck size on the proportion of cooperators. Dots represent mean proportion ± SEM of the population with WS colony morphology on day 16 of the experiment.

**Figure 2:**
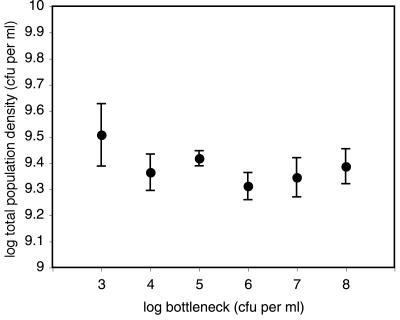
The effect of population bottleneck size on total population density. Dots represent mean ± SEM population density on day 16 of the experiment.

As predicted, the frequency of evolved SM cheats inhabiting the biofilm increased with increasing bottleneck size (Figure 1, Table 1; *F*_1,19_ = 5.72, *P* = 0.027), suggesting that reductions in relatedness caused by larger bottlenecks disfavoured cooperation. SM is also able to inhabit the broth phase of microcosms, however no significant growth in the broth phase was observed during this study (data not shown), this is in line with previous work performed under this disturbance frequency where the majority of evolved SM inhabited the biofilm [15]. There was also a significant effect of founding genotype on the frequency of evolved SM cheats (*F*_3,19_ = 4.32, *P* = 0.018), suggesting, as has been previously observed [18], that independent WS genotypes differed in their susceptibility to invasion by evolved SM cheats. It is possible that these results could have arisen through differences in population density between bottleneck treatments, however this is unlikely as there was no significant correlation between the proportion of cooperators and population density (Pearson's *r* = 0.029, *P* = 0.9). Furthermore, no significant effect of bottleneck size on total population density was observed (Figure 2, Table 2; *F*_1,19_ = 3.07, *P* = 0.096), although a significant effect of founding genotype was observed (*F*_3,19_ = 4.42, *P* = 0.016). This confirms previous work suggesting that independent WS genotypes vary in terms of productivity [18].

**Table 1:** GLM of arcsin square root transformed proportion of WS, using Adjusted SS for Tests.

Source	DF	Seq SS	Adj SS	Adj MS	F	P
population	3	0.32139	0.32139	0.10713	4.32	0.018
bottleneck	1	0.14171	0.14171	0.14171	5.72	0.027
Error	19	0.47109	0.47109	0.02479		
Total	23	0.93418				

**Table 2:** GLM of log_10_ total population density, using Adjusted SS for Tests.

Source	DF	Seq SS	Adj SS	Adj MS	F	P
population	3	0.14565	0.14565	0.04855	4.42	0.016
bottleneck	1	0.03368	0.03368	0.03368	3.07	0.096
Error	19	0.20862	0.20862	0.01098		
Total	23	0.38794				

These results extend the findings of a previous study, which highlighted the role of disturbance frequency in the maintenance of microbial social traits [15]. Here, I show that disturbance size is also important. The “intermediate disturbance hypothesis” suggests that intermediate levels of either disturbance frequency or intensity (disturbance size weighted by frequency) will favour maximal levels of diversity; both frequency and intensity are assumed to be largely equivalent in their effects, preventing domination of patches by the most competitive species [27]. It has been previously shown that intermediate disturbance frequencies favour cooperation [15]. This occurs because disturbances cause population bottlenecks that increase relatedness thus favouring cooperation. However very high frequencies of disturbance cause population densities to fall below that required for cooperation to be beneficial, while very low frequencies allow evolved cheats to accumulate. The results presented here suggest that disturbances of large effect are more likely to favour cooperation, thus for an intermediate frequency of disturbance, disturbances of greater intensity are more likely to favour cooperation because of the relatedness enhancing effects of population bottlenecks. Therefore, when considering social traits disturbance frequency and intensity may not have equivalent effects.

Ecologically generated population bottlenecks are likely to be common in nature, and thus may indeed play a key role in the evolution of microbial social traits. This work expands upon previous studies, which have used fixed [15] or very small bottlenecks [16], by using a wide range I show that the size of bottleneck generated by ecological events may be crucially important to the maintenance of cooperation in microbes. Such events may be particularly important in the evolution of microbial pathogens where it is common for single or few cells to initiate infections [28]. Furthermore, the action of the immune system and antimicrobial treatments impose further population bottlenecks that may help to maintain social traits in microbial pathogens. This is likely to be significant as social traits are thought to be important in determining both the virulence and persistence of pathogenic microbial infections [29], [30], [31].

## References

[1] Travisano M, Velicer GJ (2004). Strategies of microbial cheater control.. Trends Microbiol.

[2] Velicer GJ (2003). Social strife in the microbial world.. Trends Microbiol.

[3] West SA, Griffin AS, Gardner A, Diggle SP (2006). Social evolution theory for microorganisms.. Nat Rev Microbiol.

[4] Crespi BJ (2001). The evolution of social behaviour in microorganisms.. Trends Ecol Evol.

[5] Frank SA (1998). Foundations of social evolution..

[6] Hamilton WD (1997). Narrow Roads of Gene Land: The Collected Papers of W.D.Hamilton: Evolution of Social Behaviour Vol 1.

[7] Hamilton WD (1964). The genetical evolution of social behaviour I.. J Theor Biol.

[8] Hamilton WD (1964). The genetical evolution of social behaviour II.. J Theor Biol.

[9] Parr LA, de Waal FB (1999). Visual kin recognition in chimpanzees.. Nature.

[10] Sharp SP, McGowan A, Wood MJ, Hatchwell BJ (2005). Learned kin recognition cues in a social bird.. Nature.

[11] Taylor PD (1992). Altruism in viscous populations-an inclusive fitness model.. Evol Ecol.

[12] Mitteldorf J, Wilson DS (2000). Population viscosity and the evolution of altruism.. J Theor Biol.

[13] Wilson DS, Pollock GB, Dugatkin LA (1992). Can altruism evolve in purely viscous populations.. Evol Ecol.

[14] West SA, Pen I, Griffin AS (2002). Cooperation and competition between relatives.. Science.

[15] Brockhurst MA, Buckling A, Gardner A (2007). Cooperation peaks at intermediate disturbance.. Curr Biol.

[16] Griffin AS, West SA, Buckling A (2004). Cooperation and competition in pathogenic bacteria.. Nature.

[17] Harrison F, Buckling A (2005). Hypermutability impedes cooperation in pathogenic bacteria.. Curr Biol.

[18] Brockhurst MA, Hochberg ME, Bell T, Buckling A (2006). Character displacement promotes cooperation in bacterial biofilms.. Curr Biol.

[19] Velicer GJ, Kroos L, Lenski RE (1998). Loss of social behaviors by myxococcus xanthus during evolution in an unstructured habitat.. Proc Natl Acad Sci USA.

[20] Rainey PB, Rainey K (2003). Evolution of cooperation and conflict in experimental bacterial populations.. Nature.

[21] MacLean RC, Gudelj I (2006). Resource competition and social conflict in experimental populations of yeast.. Nature.

[22] Rainey PB, Travisano M (1998). Adaptive radiation in a heterogeneous environment.. Nature.

[23] Hodgson DJ, Rainey PB, Buckling A (2002). Mechanisms linking diversity, productivity and invasibility in experimental bacterial communities.. Proc R Soc B.

[24] Brockhurst MA, Colegrave N, Hodgson DJ, Buckling A (2007). Niche occupation limits adaptive radiation in experimental microcosms.. PLoS ONE.

[25] Spiers AJ, Kahn SG, Bohannon J, Travisano M, Rainey PB (2002). Adaptive Divergence in Experimental Populations of Pseudomonas fluorescens. I. Genetic and Phenotypic Bases of Wrinkly Spreader Fitness.. Genetics.

[26] MacLean RC, Bell G, Rainey PB (2004). The evolution of a pleiotropic fitness tradeoff in Pseudomonas fluorescens.. Proc Natl Acad Sci USA.

[27] Petraitis PS, Latham RE, Niesenbaum RA (1989). The maintenance of species diversity by disturbance.. Q Rev Biol.

[28] Maynard Smith J, Feil EJ, Smith NH (2000). Population structure and evolutionary dynamics of pathogenic bacteria.. Bioessays.

[29] Brown SP, Hochberg ME, Grenfell BT (2002). Does multiple infection select for raised virulence?. Trends in Microbiology.

[30] Drenkard E, Ausubel FM (2002). Pseudomonas biofilm formation and antibiotic resistance are linked to phenotypic variation.. Nature.

[31] Fux CA, Costerton JW, Stewart PS, Stoodley P (2005). Survival strategies of infectious biofilms.. Trends Microbiol.

